# A Rare Case of Pleural Melanoma Masquerading as a Tubercular Pleural Effusion

**DOI:** 10.7759/cureus.89742

**Published:** 2025-08-10

**Authors:** Ashwin Unni, Sanjay N Gaikwad, Rakhi A Gosavi, Lakshita pawar

**Affiliations:** 1 Pulmonology, Byramjee Jeejeebhoy Government Medical College, Pune, IND; 2 Pulmonary Medicine, Byramjee Jeejeebhoy Government Medical College, Pune, IND

**Keywords:** adenosine deaminase (ada), immunohistochemistry(ihc), pleural biopsy, pleural melanoma, pleural tuberculosis, rare cause of pleural effusion

## Abstract

This case report highlights the case of an extremely rare case of primary pleural melanoma in a 15-year-old female with left-sided exudative pleural effusion with pleural fluid adenosine deaminase (ADA) in the tuberculosis range. Melanoma is an aggressive malignant tumor mainly arising from the skin, but very few cases have been reported of a primary melanoma arising from the pleura. In addition, this patient's pleural fluid ADA was in the tuberculosis suspect range, but a detailed analysis of the pleural fluid and pleural biopsy helped in pinpointing the diagnosis. Through this case, the entire pleural fluid analysis, including the pleural fluid routine microscopy, pleural fluid cytology, and even pleural biopsy in select cases, needs to be assessed in order to avoid misdiagnosis of tuberculosis.

## Introduction

Pleural fluid adenosine deaminase (ADA) is an effective, cheap, and reliable diagnostic tool available in most laboratories to diagnose tuberculous pleural effusion (TPE). It has become a common investigation to diagnose tubercular pleural effusion. A value of more than 40 U/L is considered diagnostic for TPE, especially in a high tuberculosis prevalence country like India [1]. Other pleural fluid investigations supporting the diagnosis of TPE include elevated pleural fluid lactate dehydrogenase (LDH), elevated protein, and elevated total nucleated cell count (TNC), usually lymphocytic predominant (can be neutrophilic predominant in early stages) with less than 5% mesothelial cells. Unlike sputum Cartridge-Based Nucleic Acid Amplification (CBNAAT), pleural fluid CBNAAT has a low sensitivity [2].

Despite good specificity of 90 % and sensitivity of 92 %of pleural fluid ADA for diagnosing TPE, it can be positive in many other conditions such as malignancies (particularly lymphomas), infectious diseases (e.g., brucellosis, Q fever), and connective tissue diseases such as rheumatoid [3].

Melanoma is an aggressive malignant tumor primarily arising from the skin in more than 90 percent of the cases, but primary pleural melanoma is extremely rare, and there have been very few cases of primary melanoma lesions arising from the pleura, with only eight reported cases so far [4,5].

Through this rare case of primary pleural melanoma, an emphasis will be laid on not using pleural fluid ADA as a sole pleural fluid investigation in diagnosing TPE.

## Case presentation

Case

**Figure 1:**
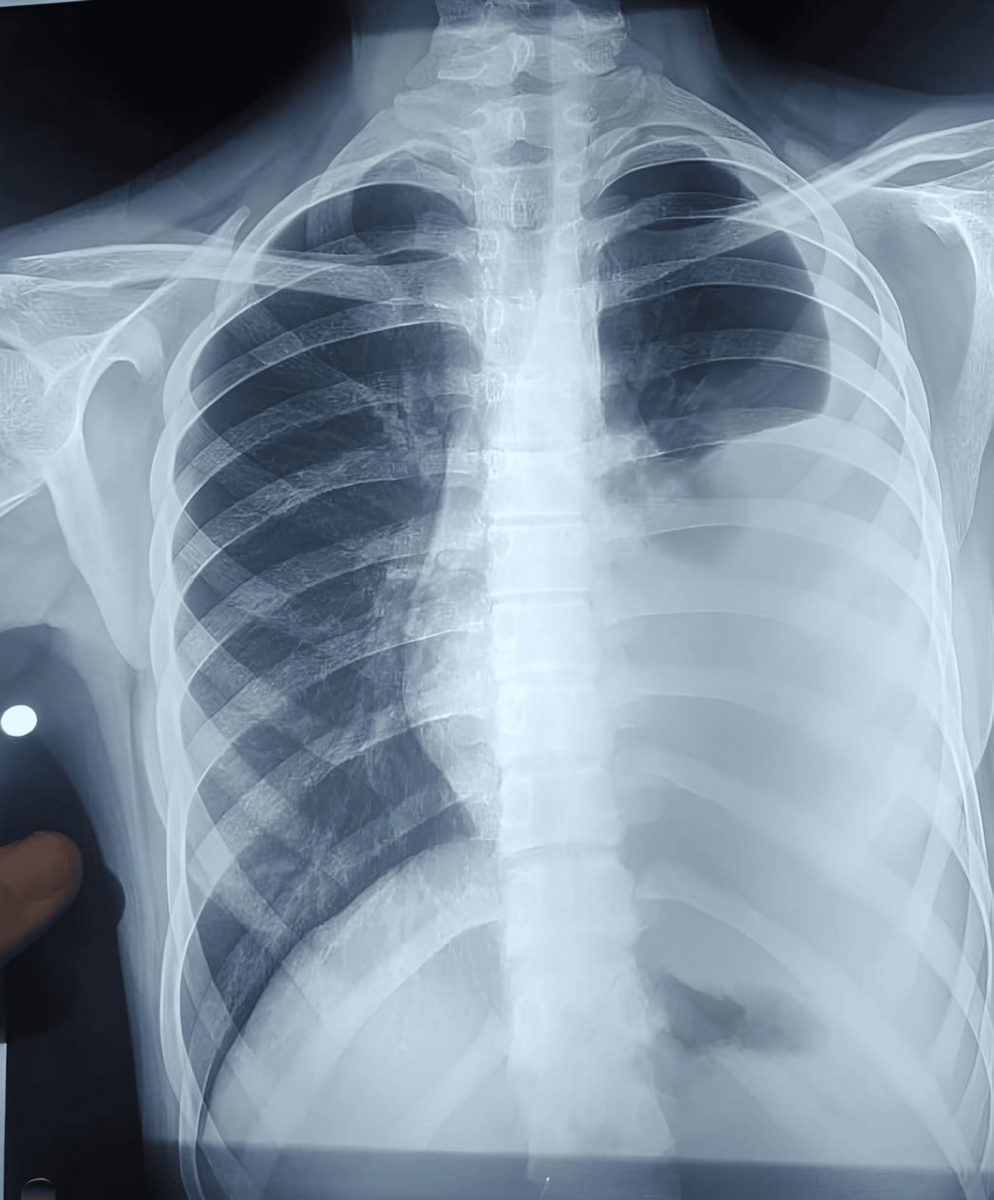
Chest X-ray showing left sided pleural effusion (pre-thoracocentesis)

**Figure 2:**
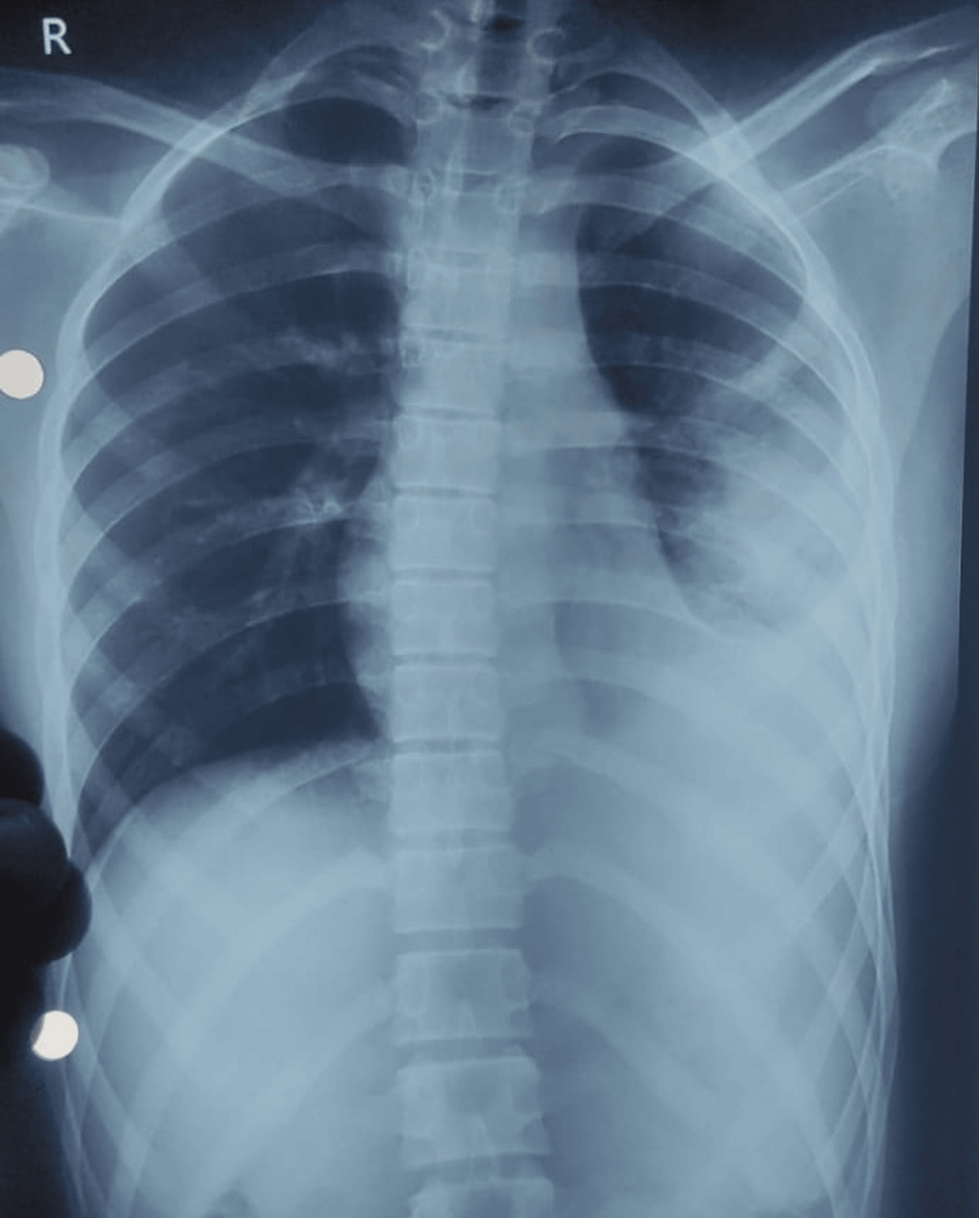
Chest X-ray showing left sided pleural effusion (post-thoracocentesis)

15-year-old female, referred from a primary health care center with chief complaints of breathlessness of exertion (progressing from modified Medical Research Council (mMRC) grade 0 to grade 4), left-sided chest pain, and generalized weakness since three weeks, with no significant co-morbidities or similar illness in family. There was no history of trauma, no history of tuberculosis contact, or previous history of tuberculosis given by the patient. She had a history of a neck swelling in November 2020, which was excised and later diagnosed as a spindle cell sarcoma, but her post-excision positron emission tomography (PET) scan showed no significant fluorodeoxyglucose (FDG) uptake. At the time of presentation, her oxygen saturation (SpO2) was 94 percent on room air, respiratory rate was 25 breaths per minute, pulse 130/minute, and blood pressure of 100/66 mmHg. She had reduced chest movements on her left side, with absent breath sounds on the left side of her chest, and reduced vocal resonance. A chest X-ray was done, which was suggestive of a massive pleural effusion on her left side. Diagnostic and therapeutic thoracocentesis was done with the due consent of her relatives, under ultrasonography guidance, after which she had symptomatic improvement and her respiratory rate and SpO2 improved. Post-procedure chest X-ray done to rule out post-procedure pneumothorax (Figures 1, 2).

Investigations

Approximately 1.2 liters of pleural fluid were drained from her left side, which was hemorrhagic in nature. Pleural fluid analysis is shown in Tables 1, 2.

**Table 1:** Pleural fluid study of the case LDH - lactate dehydrogenase; TNC - total nucleated cell count; CBNAAT - Cartridge-Based Nucleic Acid Amplification Test; Mtb - *Mycobacterium tuberculosis*; ADA - adenosine deaminase

Variable	Laboratory Finding
Colour	Red
Protein	3.94g/dl
Sugar	51mg/dl
LDH	1962U/L
TNC	1120 cells/mm3
	Lymphocytes- 20 %
	Mesothelial cells + Macrophages 80%
CBNAAT for Mtb	Mtb not detected
ADA	50 U/L
Cytology	Malignant Cells not seen

**Table 2:** Reference table showing criteria to label pleural fluid as exudative LDH - lactate dehydrogenase Source: Gautam et al. [6]

Rule	Criteria
Light's criteria	Pleural fluid/ serum LDH ratio>0.6 OR
	Pleural fluid/ serum protein>0.6 OR
	Pleural fluid LDH> two-thirds of the upper limit of normal serum LDH
Rule that does not require serum tests	Pleural fluid protein>2.9g/dl OR
	Pleural fluid cholesterol >45mg/dl OR
	Pleural fluid LDH>0.45 times upper limit of normal serum LDH

**Figure 3:**
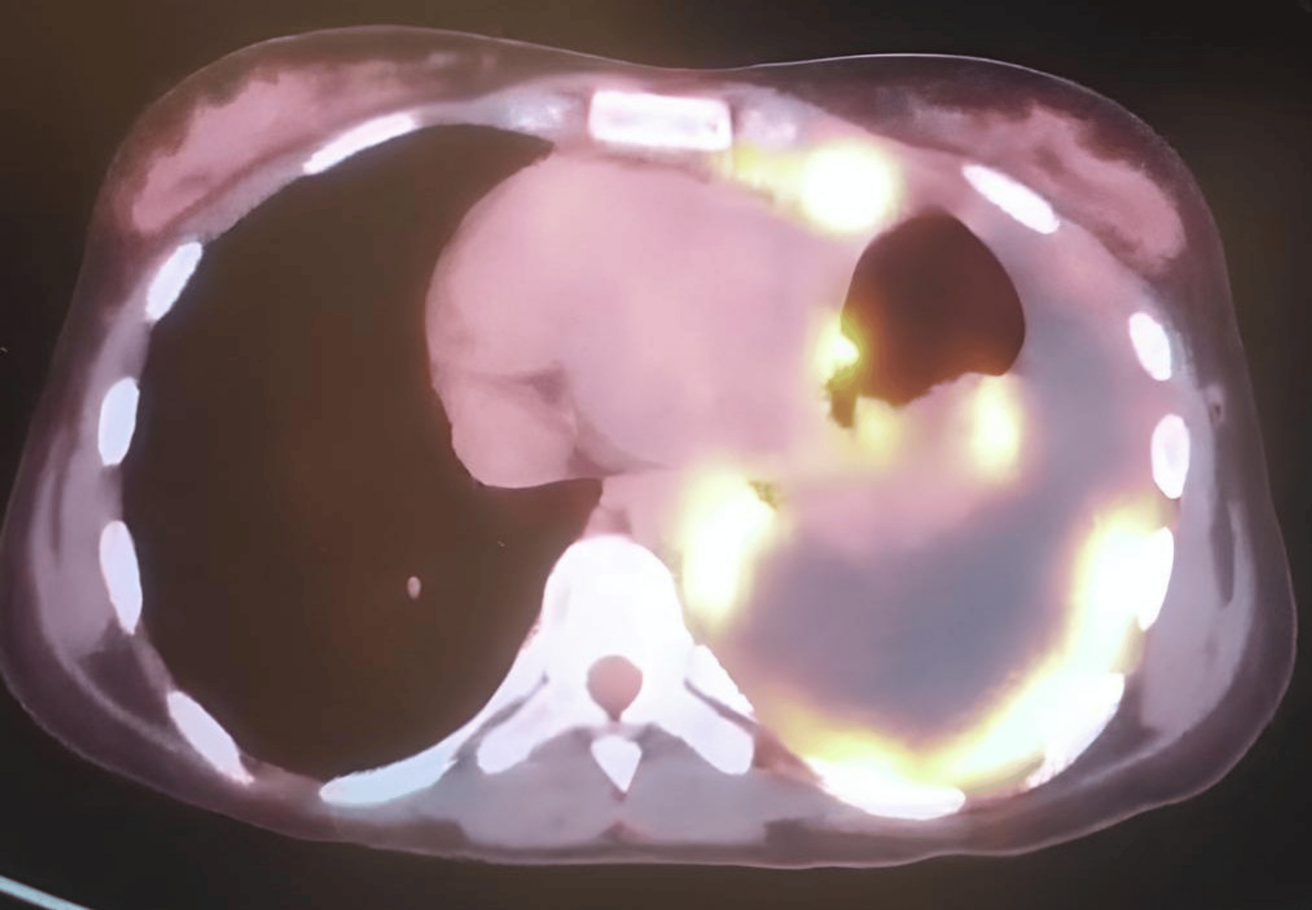
PET-CT scan showing left sided pleural effusion with left pleural enhancement PET-CT - positron emission tomography computed tomography scan

The patient's pleural fluid total protein was greater than 2.9g/dl, and her pleural fluid LDH was more than 0.45 times the upper value of normal serum LDH (normal serum LDH 140-280U/L), which fits the criteria for exudative pleural effusion [6]. Her pleural fluid cytology came negative for malignancy, but in view of her unusual pleural fluid analysis report, a PET-CT scan was done, which was suggestive of a thickened left pleura with increased FDG uptake (SUV max 15.9) with similar FDG uptake in left internal mammary and para cardiac nodes along with left sided pleural effusion (Figure 3).

This was followed by a USG-guided pleural biopsy with due consent and aseptic precautions.

**Figure 4:**
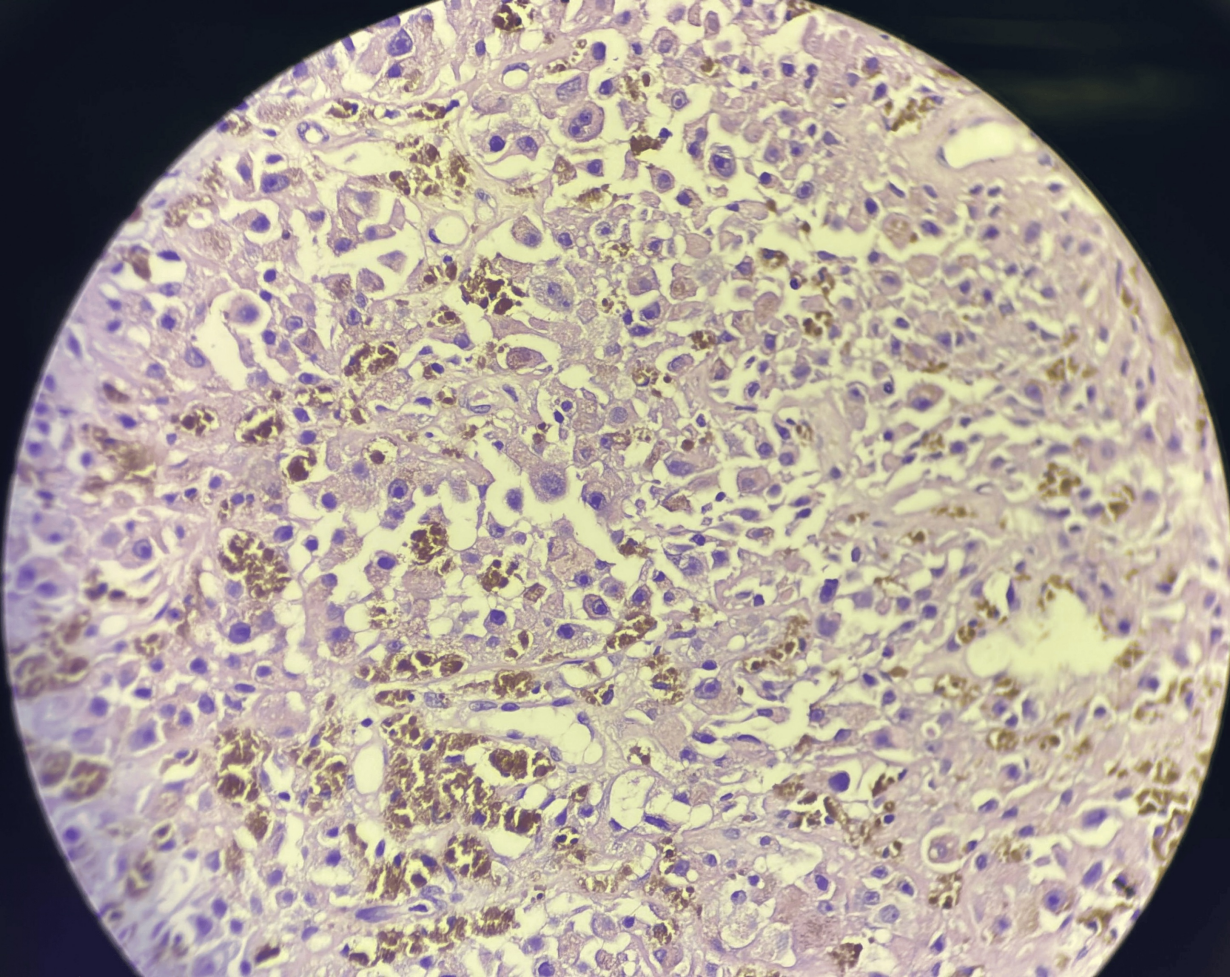
Histopathology of pleural biopsy showing sheets of atypical cells with hyperchromatic nuclei with brownish melanin pigmentation

The pleural biopsy results showed sheets of atypical cells with large hyperchromatic nuclei, conspicuous nucleoli and moderate to abundant eosinophilic cytoplasm with brownish pigment in cytoplasm along with immunohsitochemistry (IHC) positivity for S100, HMB-45 and negative result for CK and calretinin along with a Ki 67 index of 40-50%; suggestive for a metastatic malignant melanoma (Figure 4).

A dermatology screening was also done for the patient to rule out any evidence of a primary melanoma arising from any skin lesion. She was later discharged with follow-up from the medical oncology side and started on a cisplatin, dacarbazine, and vinblastine-based regimen.

## Discussion

Considering the patient's subacute presentation, exudative pleural fluid picture and a pleural fluid ADA of 50 U/L in a tuberculosis endemic country like India often warrants the diagnosis of pleural tuberculosis but in view of her hemorrhagic pleural fluid presentation and predominance of mesothelial cells in her pleural fluid routine microscopy lead to consideration for an alternative diagnosis for which a pleural fluid cytology, PET- CT scan and eventually a USG guided pleural biopsy was done to establish the diagnosis of primary pleural melanoma in this patient. Despite pleural fluid ADA being an excellent investigation for diagnosis pleural tuberculosis (sensitivity and specificity of 92% and 90% respectively), it needs to be used in conjunction with the pleural fluid analysis and microscopy to establish the diagnosis as pleural fluid ADA can be raised in conditions like malignancies (particularly lymphomas), brucellosis, Q fever and Rheumatoid Arthritis (these diseases can mimic symptoms of tuberculosis) [2,3]. This is supported by case reports by Hayashino et al. related to primary effusion lymphoma-like lymphoma (PEL-LL) mimicking pleural tuberculosis by having raised pleural fluid ADA levels [7].

## Conclusions

Through this rare case of pleural melanoma, emphasis needs to be laid on using pleural fluid ADA in conjunction with pleural fluid analysis, routine microscopy, and ancillary investigations like pleural fluid malignant cytology, PET-CT, and pleural biopsy, and not to use pleural fluid ADA alone to diagnose tubercular pleural effusions.
